# Cincinnati Homœopathy, under Allopathic Treatment

**Published:** 1850-02

**Authors:** 


*Cincinnati Homœopathy, under Allopathic Treatment.*—The following candid and fearless *expose* of Homœopathic knavery, as practised by the apostles of that system in Cincinnati, is taken from the columns of the "Methodist Expositor," of that city. It is from the pen of its talented Editor, Dr. Latta, who has in this communication done essential service to the cause of humanity, and for the bold stand he has taken against that species of quackery, deserves the thanks of the entire profession. It will be read with the deepest interest.—*Ohio Med. and Surg. Journal.*

---

### *Homœopathic Trumpet — An Uncertain Sound.*

"If the trumpet give an *uncertain* sound, who shall prepare himself to the battle."

In ancient time, the trumpet was employed for a few important purposes only. The Lord commanded Moses to make two trumpets of beaten silver, to call the people together, when about to decamp: see Numbers, chap x. They were also used to proclaim the beginning of the civil and Labbatical years, and the commencement of the year of jubilee: Leviticus, chap. xxv.

At first there were but two trumpets; but in after times they were greatly multiplied. In the days of Joshua there were seven trumpets. At the dedication of Solomon's temple, six score priests sounded as many trumpets. At still later periods they have been employed for war purposes. While in modern times, it seems, dinner horns and common newspapers have been substituted, for the purpose of claiming public attention. And, indeed, many of our own day have become their own trumpeters, making, often, very uncertain sounds. As a remarkable instance of this sort, we invite attention to an extract from the bulletin of Doctors J. H. Pulte and B. Ehrmann, of this city, that appeared in the Daily Times, of the 13th inst., which is certainly the loudest blast of the trumpet that has been sounded since the falling of the walls of Jerico, where ram's horns were employed. Indeed, it is possible that the scripture incident had a powerful influence on these homœopathic gentlemen, in the selection of a trumpet, as a medium of communication; but still we fear that their trumpet has given an uncertain sound. Their reports of cholera and of cures are so extravagant, that few are disposed to believe them, while thousands in this community unhesitatingly pronounce them false.

The following is the extract from their report:

"We have treated," says Drs. Pulte and Ehrmann, "from the 1st May to the 1st August inst., 1,116 cholera patients, of which 538 exhibited the symptoms of vomiting, diarrhœa, and cramps, including a great many, from 90 to 70, in deep state of collapse—the balance, 578, had the symptoms of vomiting and rice-water discharges, and were prevented from running into a higher stage of the disease by early applications of the proper medicines.

"Of the collapse cases, a great many were cured, the success depending upon the medicines given in the early stages. In those improperly treated, by opiates particularly, our success was difficult; but in cases where the patient was treated at first, by camphor alone, or where he went immediately into collapse, after being attacked, the result was very favorable.

"Of the 1,116 cholera patients, 474 were Americans, and 642 Germans, including a few Irish; the mortality of the whole number was 35, of which two were Americans and 33 Germans. Of the latter not one-half should have died, but from their carelessness of diet, and want of knowledge of the insidious character of this disease. We counted among those who died, all of which we had attended ourselves, even if we were called at too late a time to be of real use.

"Besides the above 1,116 cholera patients, we treated, during the same time, 1,350 cases of a mixed character, mostly diarrhœas with rumbling in the bowels, (cholerina,) and towards the close of the epidemic, a great number of dysenteries, some of which were of a very malignant character, (we lost none of them, however,) also a good many nervous fevers with typhoid tendency.

"To verify the above statement, we have made out a complete list of all the cholera cases, with names and dates, for reference, at any time when required.

"The principal remedy used in the beginning of cholera was camphora, the tincture of which was prepared in the proportion of one part of the gum to six parts of alcohol, as advised by Hahnemann himself, who first recommended this remedy in 1829. The dose in which it was applied, was equal to one or two drops every five minutes, for one or one and a half hour, until profuse perspiration ensued. During this time, the patient had to be well covered, and in most cases the camphor alone produced a complete cure, without the help of any other remedies.

"If, however, it did not, because the second stage of the disease had appeared, veratrum and cuprum were used, especially against cramps, also secale cornutum, (ergot,) particularly in elderly individuals; and in cases of collapse, carbo (vegetabilis coal,) and arsenicum, the two latter in the 30th dilution."

In noticing the above, we do not design to discuss the merits of homœopathy as a system of practice. This is not the province of a religious journal. It is the moral, and the propriety of the report with which we have to do. Professional men, above all others, are expected to conform to the rules of propriety and morality, and in both of these respects we think the above is at fault. First, it is undignified and unprofessional to appear in the public prints in praise of one's self, and a regular physician who would do it, would not be respected or recognized by the profession. It is a method adopted by quacks, and nostrum sellers, and has always been looked upon with contempt by professional men. And to say no more, it is so immodest that we doubt whether an American could make a report of this sort, without incurring the universal disapprobation of the community; and it has yet to be tested whether the community will tolerate this kind of outrage upon the rules of propriety, even by foreigners—

Germans, who, for the sake of gain, thus rudely attempt to sound their own trumpet in the public ear.

We object to the above report of Drs. Pulte and Ehrmann, secondly, because it is immoral.

*First,* They profess to have been practising homœopathy, for the cure of cholera and other diseases, when, in fact, according to their own showing, they have adopted the allopatic treatment universally. The principal remedy employed, according to their own statement, was the strongest tincture of camphor in a dose of one or two drops every five minutes; and in some instances, we have known the homœopathists to administer from three to five drops every three minutes, which was equal to from fifteen to twenty grains of camphor every hour. Now it is known to every regular physician, that this is a total abandonment of the principles of homœopathy.

"*Similia similibus curantur:*" that which will produce the disease will cure it, is the great fundamental principal upon which the system is founded. Had they acted in harmony with this pretension, they wonld have given to their cholera patients something which would have produced purging and vomiting, such as ipecac, tartar emetic, etc. But alas, instead of this, we find them employing camphor, and that, too, in larger doses than it is administered by most of their allopathic neighbors. But who, we ask, ever heard that camphor was emetic and cathartic.

The infinitesimal doses, as well as the fundamental principle, according to the showing of Drs. Pulte and Ehrmann, have been abandoned, and yet they ascribe their cures to homœopathy. We doubt whether they will succeed in gulling the intelligent in the community much longer by a system of quackery so palpably absurd—so grossly immoral. We have no doubt that camphor, administered in ten or twenty grain doses, would secure a reasonable share of success, whether employed by homœopathic or allopathic practitioners. It is known to community, that regular physicians have always relied upon the use of camphor in this disease to a great extent, in much smaller doses than those prescribed by the homœopathists, and hence if the latter have been successful, it is obviously, (if their own statements can be relied upon) by the use of allopathic remedies, and not by infinitesimal doses of medicines, as they would have it understood. These gentlemen seem to have abandoned Hannemann's theory, that "the hair of the dog would cure the bite."

It is grossly immoral, we think, to practice such a deception upon community. We have long believed that homœopathic doctors were practising allopathy in disguise—employing the "sampsons" of the system, such as calomel, corrosive sublimate, arsenic, camphor, belladona, pulsatilla, and many other powerful articles, in full doses—but now we have proof which sets the question forever at rest.

It is also notorious, that during the progress of the cholera, these gentlemen homœopathists have been equally unfortunate with the regulars, in producing salivation, and of this we shall furnish proof whenever called for. Calomel, it may be, was not the agent generally employed; corrosive sublimate being a more powerful agent, and capable of solution, was preferred, and this we have found at the bedside of the sick, more than once during the prevalence of the epidemic in this city.

Heretofore we have been disposed to pity, rather than censure, some of those engaged in the practice of homœopathy, believing them the dupes of a theory the most ridiculously absurd; but to our surprise and mortification, we find that we, rather than they, were duped by the false pretensions of those who practice it. For, instead of giving inflnetesimal doses of medicines, as we supposed, which would produce the disease for which they were prescribed, we find them adopting the very same treatment employed by the regular profession. In this, we confess, we have been prodigiously gulled by these pretenders, and most cheerfully award to them a degree of cunning more than equal to the moral of the transaction.

*Second*, We object to the report of Drs. Pulte and Ehrmann, because it is immoral in its statement of facts. They affirm that they have treated four hundred and seventy-four cases of cholera among Americans since the 1st of May, and that but two out of the whole number have died. If this were true, as above remarked, the glory would not redound to homœopathy, as these gentlemen would have it, but to allopathy—to regular remedies, in full doses, as they themselves have made manifest in the report now under consideration. But alas for both systems, the report is not true.

We know not what number of cases they may have had; but that more have died than are reported by these gentlemen, is absolutely certain. In the range of our observation and acquaintance, not less than nine, instead of two Americans, are reported to have died in their hands, which is probably not one-tenth of the whole number they have lost. In making this statement, we speak advisedly, in that we have had these cases reported to us by responsible individuals, giving the names and residences of the Americans who have died under their treatment, whose names and residences will be given, if this statement should be contradicted by the persons concerned.

Now, if these homœopathic doctors are so inaccurate in their reports of cures, what reliance can be placed upon their statements in any case in which their interests are involved? Who can believe their representations either with respect to their mode of treatment or their success?

We can scarcely conceive of a higher degree of immorality than that of deceiving community, with respect to the best means of preserving their health and their lives, and yet this seems to have been the part acted by the homœopathic doctors.

We regret exceedingly, that we are called upon to make this *expose;* but, as public journalists, we feel that we could not do otherwise, without a criminal neglect of duty. For if nine cases have come within the range of our own observation, and those with whom we are associated, it is fair to conclude that the mortality attending the practice of these gentlemen is ten if not twenty times as great as they have reported it. But as we are not personally cognizant of all the facts stated, and as it is possible that some mistakes may have been made by our reporters, we shall most cheerfulty permit the said Drs. Pulte and Ehrmann to be heard in self-defence, in our columns. with respect to any fact stated in this article.

The God whom we serve knows that we would not do them injustice in any respect, and we are therefore willing to allow them the privilege proposed above, at the same time assuring them, and all others, that if subsequent events or facts should prove that we are in error, we will most cheerfully correct it.

Meanwhile we shall expect to learn more as to the results of their practice, both as it respects Americans and Germans, which it may not be necessary to publish, should our present statement not be contradicted. But in the event of contradiction, either directly or indirectly, we assure all concerned, that the na nes and residences of those who have been alluded to, as having died under their treatment, will be forthcoming, with the names and residences of all others who may be reported hereafter.

Thus far the medical profession have kept silent; but really this last attempt of the homœopathists and others, to make them responsible for the thousands who have died during the epidemic, is beyond endurance; when in truth their accusers, of all others, have been least successful, especially the homœopathists, who have been crippled in the use of regular allopathic means, by attempting to conceal them.

We have reported above, nine cases of American patients who have died in their hands, on what we conceive to be reliable authority, while, in fact, we have no doubt ninety-nine Americans and more have fallen victims to the cupidity of these distinguished homœopathists, while hundreds, if not thousands, of Germans have perished by relying upon two and a half and five dollar boxes of cholera preventives, which these gentlemen induced them to believe would be all that was needed to save them from its ravages. But of this we may have occasion to speak hereafter.

---

*New Preparation of Morphine.*—At a meeting of the "Suffolk District Medical Society,' Dr. Fisher called attention to a new preparation of morphine, with which he is at present experimenting. He dissolves morph. sulph. 2 grs. in 1ʒ of chloroform; ten drops, inhaled by the mouth, in cases of phthisis, will give immediate relief to the harrassing cough, and sleep follows, which lasts from an hour to an hour and three quarters. More largely administered, it checks diarrhœa in phthisis, and in doses of from ten to twenty drops, restrains the action of the bowels in dysentery.—*Boston Med. and Surg. Journal.*

